# Pericardial agenesis - the wandering heart

**DOI:** 10.1186/s43044-023-00405-x

**Published:** 2023-09-19

**Authors:** Tushar Kalekar, Latha P. Reddy, Deepak Koganti, Nikhith Soman

**Affiliations:** Department of Radiology, Dr. D.Y. Patil Medical College, Hospital and Research Centre, Sant Tukaram Nagar, Pimpri Chinchwad, Pune, Maharashtra 411018 India

**Keywords:** Pericardial agenesis, Cardiac MRI, Chest X-ray, Myocardial infarction

## Abstract

**Background:**

Congenital pericardial absence is an uncommon cardiac anomaly that is typically asymptomatic and commonly misdiagnosed due to a lack of symptoms or atypical symptoms. Pericardial agenesis (PA) should be considered one of the differential diagnoses when the patient presents with chest pain. This case shows how the diagnosis of pericardial agenesis is made exclusively using multi-modality imaging, starting from findings in a basic chest radiograph to cardiac MRI, while also demonstrating the classic signs seen in this condition. Magnetic resonance imaging of the heart is the gold standard for determining the absence of pericardium in the prognosis.

**Case presentation:**

A 32-year-old male who presented with chest discomfort and radiating pain to his back and left shoulder mimicking myocardial infarction with normal ECG and enzyme markers. A chest radiograph (taken 24 h apart) demonstrates the left lateral position of the heart and the bulging contour of the left heart border, a lucent area between the aorta and pulmonary artery. Subsequently, cardiac MRI reveals left pericardial agenesis.

**Conclusions:**

This article provides insight into a rare differential to consider in a young patient presenting with chest discomfort. This case shows how the diagnosis of pericardial agenesis is made exclusively using multi-modality imaging, starting from findings in a basic chest radiograph to cardiac MRI, while also demonstrating the classic signs seen in this condition.

## Background

Pericardial agenesis is an infrequent heart anomaly that can be partial or complete [1, 2]. Defective development of the pleuro-pericardial membranes that link the pericardial and pleural compartments leads to whole or partial pericardial absence, with partial deficiency being more common than complete absence [3, 4]. The pericardium is a fibrous sac that surrounds the heart and provides support and protection. Its absence can occur in certain medical conditions, such as congenital absence or surgical removal. Up to 70% of cases are caused by total amputation of the left pericardium [5]. While the majority of people lacking a pericardium are asymptomatic, they may have a variety of clinical symptoms. However, cardiopulmonary disease (CPD) is frequently unconnected to other cardiovascular or pulmonary congenital defects. Males outweigh female’s three to one, and left-sided abnormalities account for the majority (86%) [6]. It is important to note that the absence of the pericardium alone may not always cause significant clinical problems. In some cases, individual patients can live without symptoms or complications. However, in others, it may be associated with certain cardiac abnormalities or functional changes that require further evaluation and management. Radiological imaging plays a crucial role in assessing these aspects and guiding appropriate medical interventions if necessary.

We describe an unusual example of a total absence of left the pericardium accompanied by an atrial septal defect of secundum type (ASD).

Technique: Multiplanar cardiac magnetic resonance imaging was done. Acquisitions were made in four-chamber, short-axis, and two-chamber perspectives.

## Case presentation

A 32-year-old male driver by profession presented to the cardiology department complaining of chest discomfort and a history of pain radiating to his back and left shoulder, which had gotten worse following four days of weight training. There are no comorbidities. History of similar complaints in the past with the first episode at the age of 10 years and similar such complaints are noted on lifting heavy weights, climbing uphill and on exertion. There is no history of concomitant surgical operations or substance abuse. There is no family history of ischaemic heart disease. Clinical assessment is normal. Routine blood tests and cardiac markers are normal. On presentations, vitals were heart rate of 164 beats per minute (tachycardia), blood pressure of 130/84 mmHg, and respiratory rate of 24 cycles per minute (tachypnoea), with normal blood glucose sugar level and showing normal ECG findings.

**Fig. 1:**
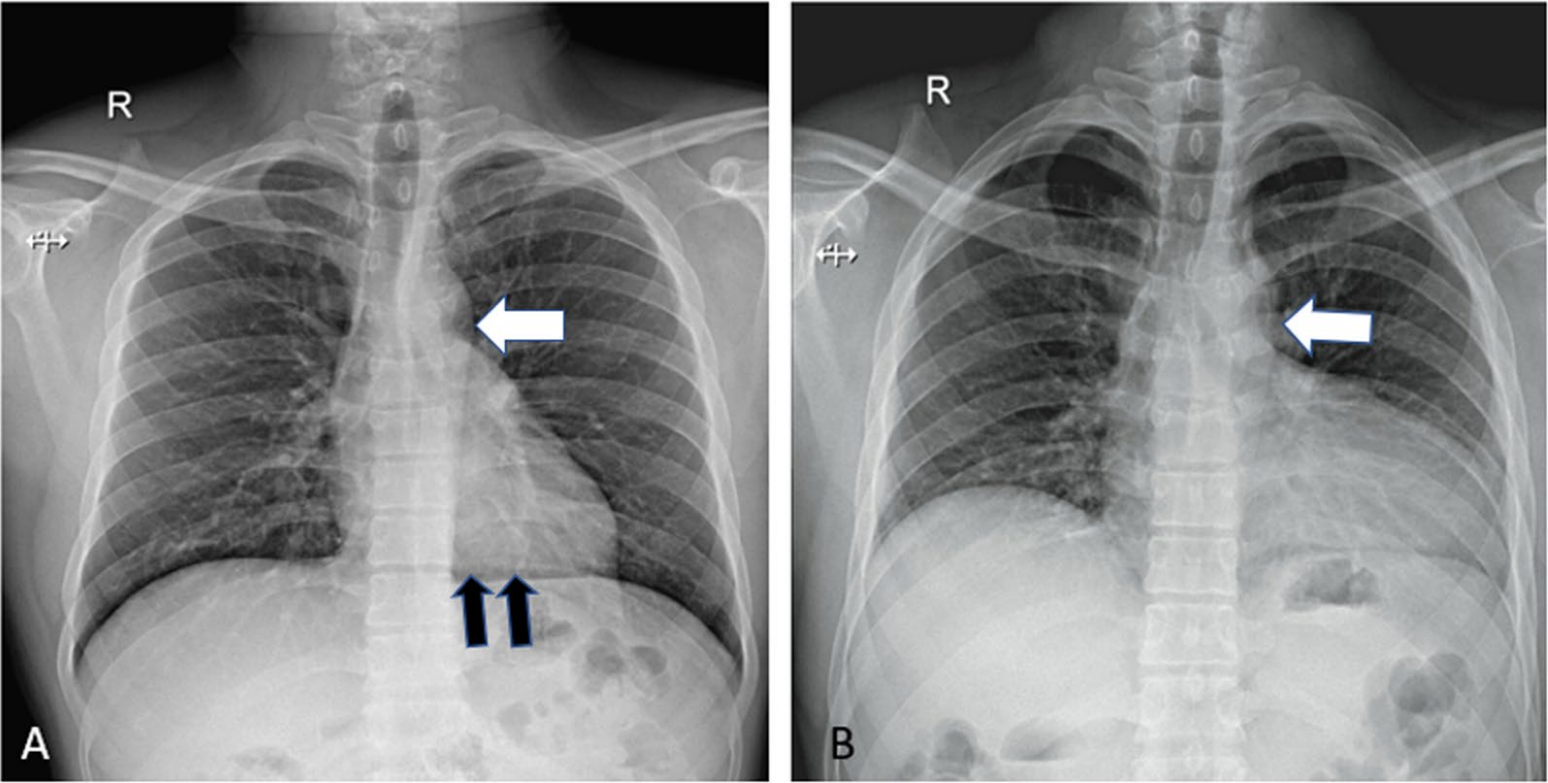
**A** Chest radiograph reveals normal position of left heart border and apex of heart with and a lucent area between the aorta and pulmonary artery (white arrow). **B** Chest radiograph (taken after 24 h) demonstrating the leftward position of the heart and the bulging contour of the left heart border lucent area between the aorta and pulmonary artery (white arrow). Lucency between the left hemidiaphragm and the base of the heart (black arrows)

**Fig. 2:**
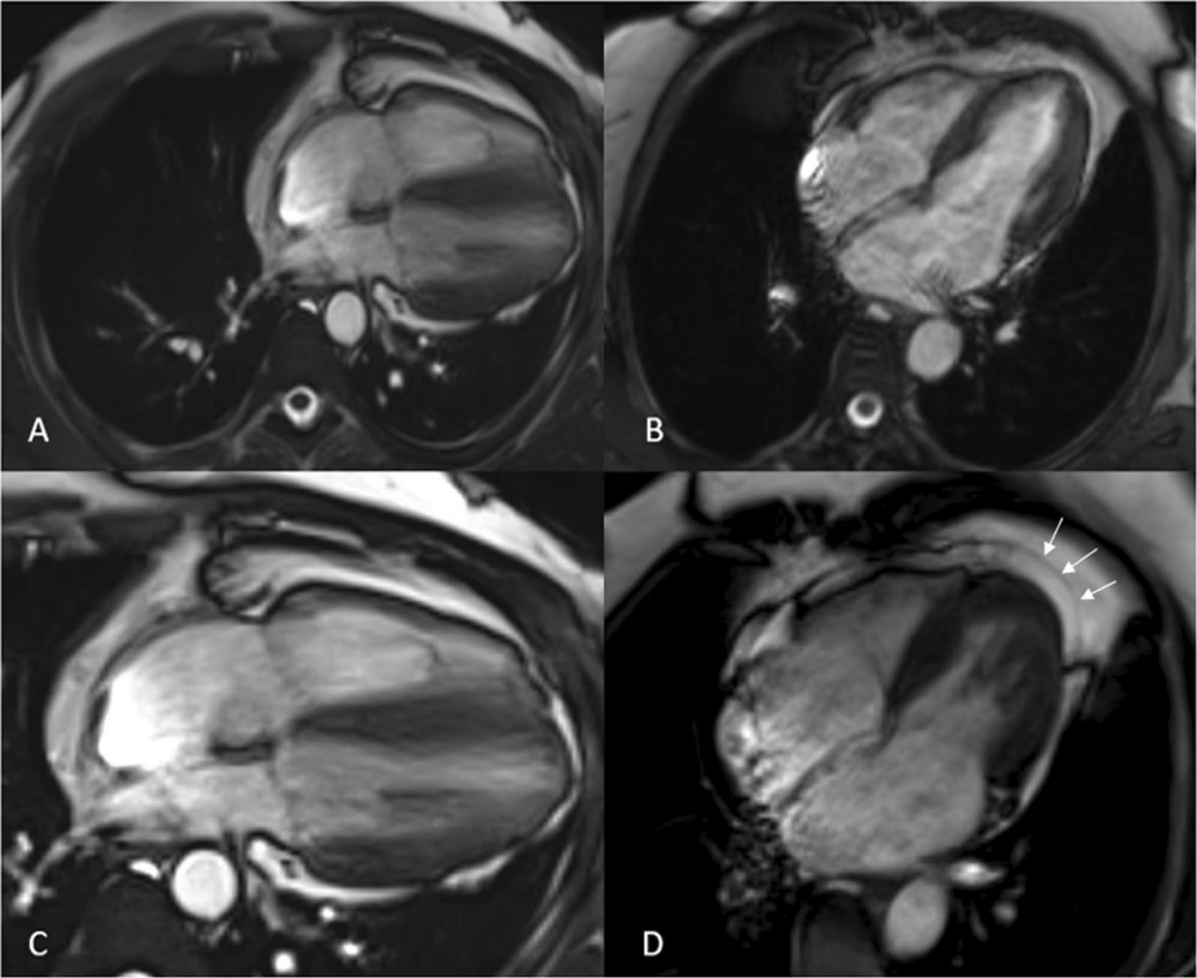
Images seen are comparisons of pericardial agenesis (left) with the normal cardiac MRI (right) for better understanding. Image **A** CINE image of four-chamber view showing excessive levorotation of the heart in comparison to the image **B** which is of normal patient showing normal location of the heart. Image **C** CINE image of four-chamber view reveals non-visualization of pericardium in the apical and posterior cardiac margins, which is clearly seen in the image **D** revealing normal cardiac MRI marked with white arrows

The patient was directed to the radiology department for additional assessment. The chest X-ray indicated a normal apex and left heart border but a lucent region, as well as lung tissue between the left hemidiaphragm and heart base (Fig. 1A). The patient was referred for echocardiography, which revealed substantial cardiac mobility with a high degree of levorotation, normal left ventricle size, normal systolic function, and a 60% ejection fraction. The next day, the patient was suggested to undergo a cardiac MRI, which revealed significant levorotation of the heart (Fig. 2A) and lung tissue interposition anteriorly between the main pulmonary artery and aorta. An analogous interposition of lung tissue occurs between the left ventricle and the diaphragm. The left atrial appendage protrudes outward along the lateral face of the aortic arch. A 6-mm defect in the middle atrial septum is seen, most likely of the secundum type with left-to-right blood shunting. Hence, a suspicion of pericardial agenesis was made.

**Fig. 3:**
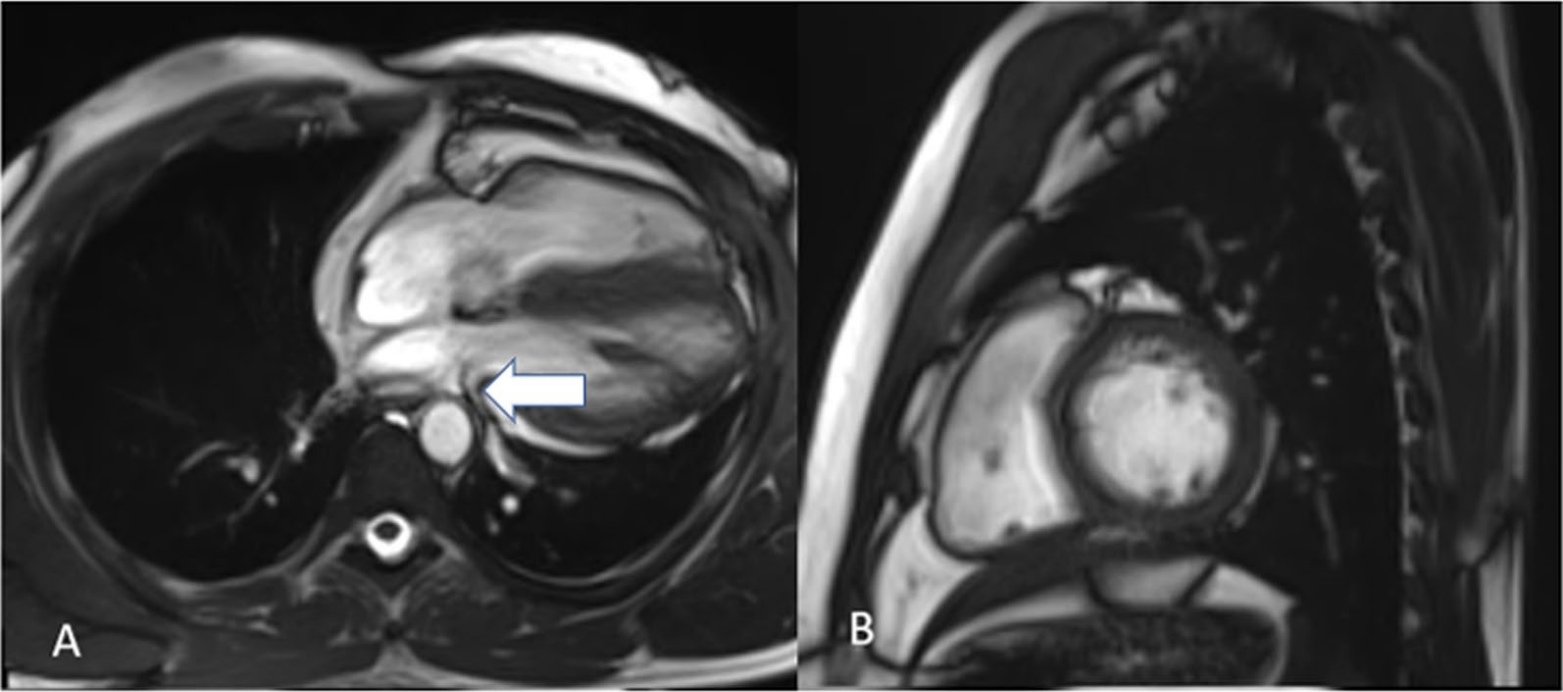
**A** Four-chamber Trufi CINE showing narrowing of calibre of left inferior pulmonary vein due to compression between the descending aorta and the left atrium, caused by excessive levorotation indicated with white arrow. **B** Short-axis Trufi CINE sequence

The left inferior pulmonary vein seems to have a restricted calibre as a result of compression between the descending aorta and left atrium, most likely caused by significant levorotation (Fig. 3A). On the left, the superior and inferior pulmonary veins measure 7 mm superior to the inferior and 4 mm inferior to the superior. On the right side, they were 18 mm and 12 mm.

**Fig. 4:**
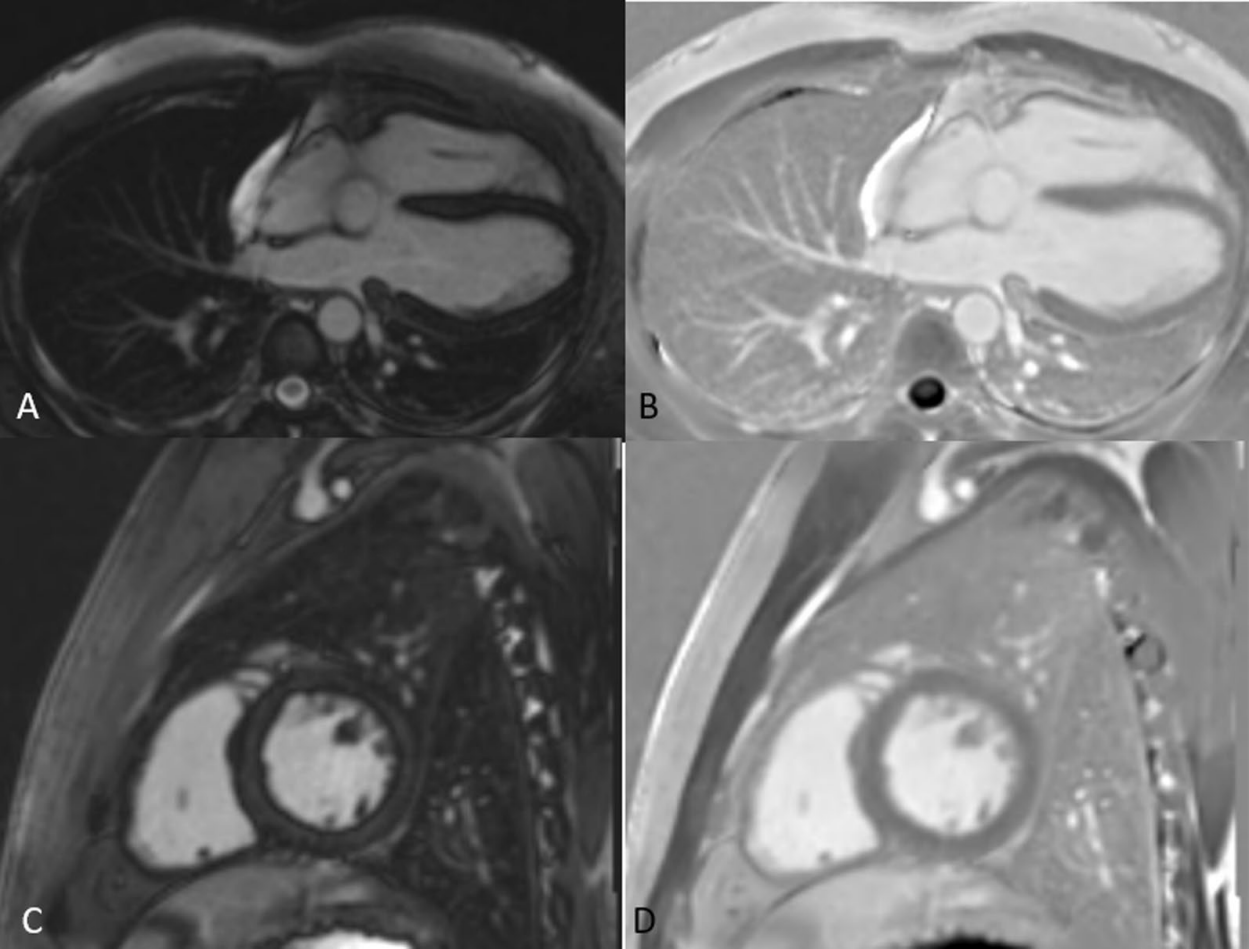
PSIR MAG and PSIR axial and short-axis images show normal signal intensity of the ventricle walls

Investigations on the mobility of the walls of the left and right ventricles show excessive cardiac mobility is defined by a 1.5-to-2 mm displacement of the heart apex. The normal pulmonary artery diameter is 25 mm. The rest of the heart looks normal. Pleural or pericardial effusion is absent with normal ventricular walls (Fig. 4).

**Fig. 5:**
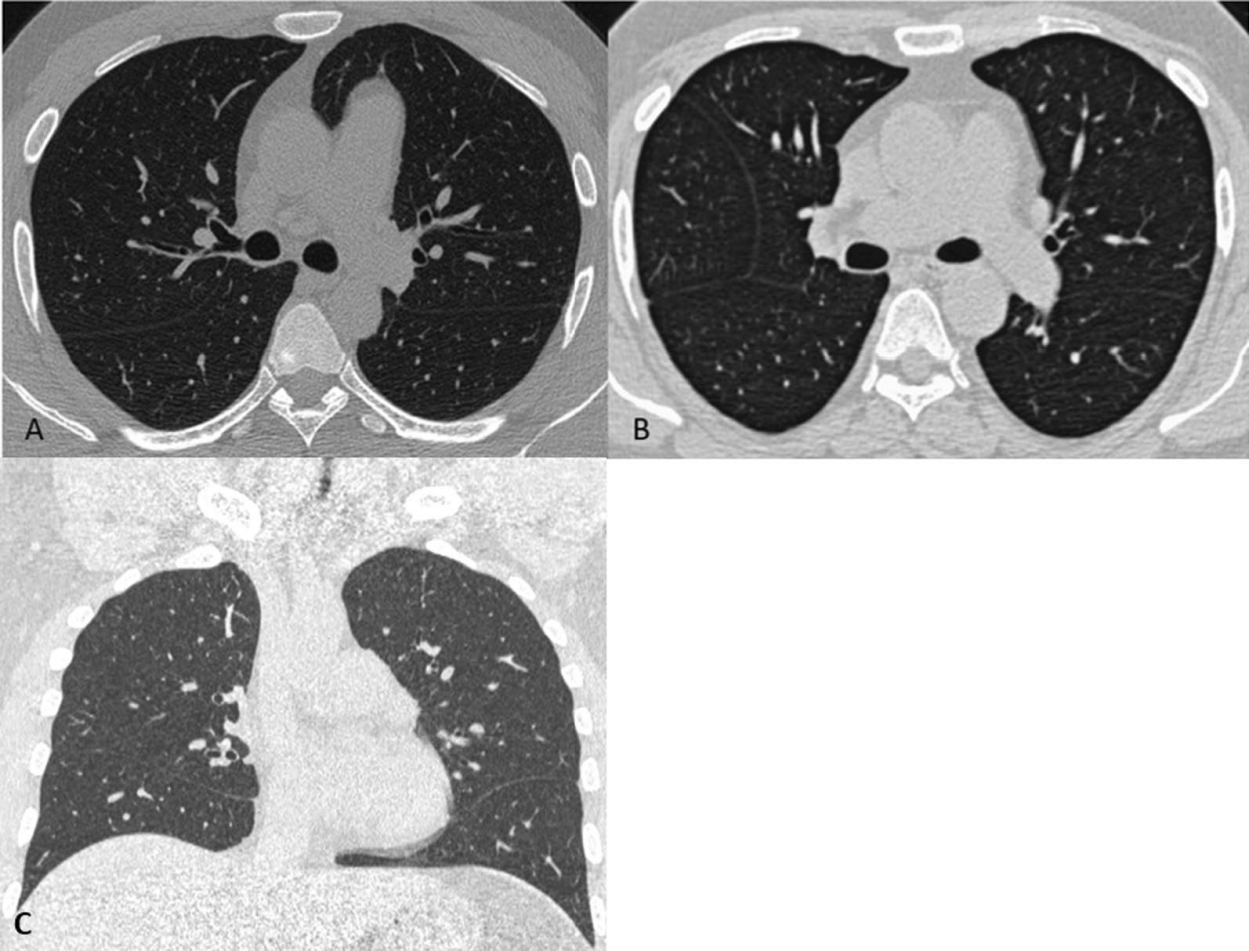
CT axial images seen are comparisons of pericardial agenesis (right) with normal CT chest (left). **A** In the absence of the pericardium, lung tissue can be detected between the aorta and the main segment of the pulmonary artery. **B** Normally, the aortopulmonary window is covered by pericardium and contains some fat. **C** Lung window—CT coronal image reveals an area of lung parenchyma between left hemidiaphragm and the base of the heart

A chest HRCT was performed, revealing the absence of the pericardium on the left side. Lung tissue may be recognised between the aorta and the primary pulmonary artery (Fig. 5A). Lung tissue was observed between the left hemidiaphragm and the heart's base, as well as an abnormally expanded left atrial appendage. The following day, with the same concerns, a repeat chest X-ray revealed a leftward migration of the heart apex (wandering cardiac apex) (Fig. 1B). The patient was treated conservatively with regular follow-up in the outpatient clinic and is advised not to lift heavy weights.

## Discussion

The pericardium is a double-layered fibro-serous sac that surrounds and stabilises the heart within the thorax, preventing it from overfilling, minimising friction, and protecting it from infection by neighbouring organs. When pleuro-pericardial membranes merge in the fifth week of embryonic development, the pericardium forms. Failures of this process, which may be caused by early Cuvier duct atrophy, result in congenital abnormalities such as complete or incomplete pericardial agenesis [7]. Inadequate blood supply to the pleuro-pericardium as a result of left common cardiac vein atrophy results in abnormal development of the pleuro-pericardial membranes and pericardial abnormalities early in life [4]. The most common ailment (70%) is the absence of the left pericardium, common in men. Complete pericardial aplasia (9%) is less common than right pericardial aplasia (17%) and left pericardial aplasia (7%). While the majority of individuals with congenital pericardial absence have isolated issues, around 1/3 of these children have other congenital cardiovascular and pulmonary disorders. Furthermore, these patients may have one or more of the following syndromes such as VATER (vertebral deformities, anal atresia, tracheoesophageal fistula, and radial and renal abnormalities), Marfan's syndrome, or Pallister–Killian syndrome (tetrasomy 12p). It is clinically recognised by a range of symptoms, including non-exercise-induced lancinating chest discomfort, disorientation, dyspnoea, and palpitations [4]. Chest radiography reveals a significant left lateral displacement of the heart profile without tracheal deviation, as well as a flattened and extended left ventricular border. Between the diaphragm and the heart, or the aorta and the pulmonary artery, intervening lung tissue forms a lucent region [8]. Frequently, the electrocardiogram displays bradycardia with right bundle branch block (as in our patient). Additionally, poor R wave progression as well as large P waves may be observed. Echocardiography is a type of cardiac imaging that can first provide information on the prevalence of complete pericardial agenesis [9]. During systole, the ventricular septum might shift paradoxically or flatly while maintaining normal systolic thickness, cardiac hypermobility, and an expanded RV. Additionally, echocardiography may be used to emphasise the LV's teardrop shape, bulbous ventricle, and extended atria in the apical four-chamber view [10]. Additionally, as observed in the two-chamber picture, the atrioventricular groove is significantly angulated, and the inferior wall of the left ventricle bulges outward [11]. Exercise–stress echocardiography may indicate an amplification of the heart's "pendulum-like" motion. Doppler methods may detect a drop in systolic flow and a decrease in the systolic-to-diastolic flow ratio in the pulmonary veins, most likely as a result of the lack of negative intrapericardial pressure [5]. Echocardiography, like chest X-rays, is frequently uninvolved in patients with incomplete/partial pericardial agenesis. Ventricle enlargement, herniation of atrial appendages, irregular ventricular wall motion, and ventricular abnormalities linked with coronary compression can all be recognised in some circumstances [9]. Imaging such as CT and MRI can be used to assess the extent of the defect (complete or partial), the presence of herniated structures, and the presence of further flaws. MRI is an excellent tool for detecting both partial and total pericardial agenesis. The normal pericardium is a small linear band surrounded by dense epicardial adipose tissue, moderately intense heart tissue, and surrounding adipose tissue. However, in 10% of people, a small amount of adipose tissue obscures the pericardium. The pericardium is more apparent above the right ventricle in systole.

During diastole, the normal pericardium is between 1.7 + 0.5 mm and 1.2 + 0.5 mm thick. Although not all pericardial recesses are visible on MRI, several are including the transverse pericardial sinus, which is dorsal to the ascending aorta, and the oblique pericardial sinus, which lies behind the left atrium. Additionally, an aberrant posture of the heart, bulging of the major pulmonary artery, and lung tissue interposition between the left hemidiaphragm and the inferior side of the heart, as well as between the great arteries, are all symptoms of a total left defect. Additionally, a herniated heart can be caused by a pericardial defect, evident in a partial defect [3]. CT helps in detecting interposed lung tissue between the major pulmonary artery and aorta window, which is considered pathognomonic for this condition, as well as a prominent main pulmonary artery [12]. On decubitus CT, complete defects display a rightward axis shift, which aids in the identification of entire and partial flaws [13]. By contrast, thoracoscopy is the recommended confirmatory procedure [3].

Patients with a full pericardial defect frequently have a favourable prognosis and are not treated until significant symptoms develop. However, in patients with partial pericardial agenesis, herniation of the heart due to tension on the chordal tissues can result in tricuspid regurgitation, catastrophic myocardial strangling, ischaemia, and sudden cardiac death. Unless complications arise, minor or full anomalies do not generally require repair. When herniation develops or is imminent in partial defects, the defect can be widened surgically by pericardiectomy or pericardioplasty [14]. Additionally, if the apical appendage is acutely strangled, a left atrial appendectomy may be considered [15]. Reconstruction is not suggested for a large defect such as the one in our instance, as the heart adapts to deformed architecture in general, and rectification attempts may result in unstable flow patterns [16]. The majority of patients with a complete defect have a reasonable chance of healing and only require surgery if complications or uncontrollable symptoms emerge [4]. Recommendations given to patients with pericardial agenesis depend on the individual’s specific symptoms, associated conditions, and overall health. However, here are some recommendations that may be considered. (1) Regular cardiac monitoring, they may need regular check-ups, electrocardiograms (ECG/EKG), echocardiograms, and cardiac imaging tests to evaluate the function and detect any potential complications. (2) Lifestyle modifications include adopting a healthy lifestyle with mild regular physical activity, weight management, avoidance of smoking and consumption of alcohol, and managing other health conditions such as high blood pressure or cholesterol levels. (3) Symptom management for some individuals with pericardial agenesis may experience symptoms like chest pain and breathlessness, which are managed symptomatically at times with the help of a healthcare professional. (4) Monitoring the complications for individuals with pericardial agenesis may be at risk of certain complications like arrhythmias or cardiac herniation. Monitoring for these complications and managing them appropriately is essential.

## Conclusions

Pericardial agenesis, although rare, should be considered in patients presenting with angina or discomfort radiating to the left arm. It can, however, be asymptomatic and have a varied range of symptoms. The present patient was incidentally diagnosed with pericardial agenesis at the age of 32. Cardiac MRI helps in knowing the type, axis, and other associated cardiovascular abnormalities.

## Data Availability

All data generated or analysed during this study are included in this published article.

## Abbreviations

ACS: Acute coronary syndrome
ECG: Electrocardiogram
MRI: Magnetic resonance imaging
PA: Pericardial agenesis
CPD: Cardiopulmonary disease
ASD: Atrial septal defect
CT: Computed tomography
LV: Left ventricle
RV: Right ventricle
HRCT: High-resolution computed tomography
PAH: Pulmonary artery hypertension
TR: Tricuspid regurgitation
